# Introducing “Visual bibliographies” as a novel tool for communicating complexity: a knowledge translation case study from Aboriginal and Torres Strait Islander primary health care research

**DOI:** 10.1186/s12913-025-13936-7

**Published:** 2026-01-23

**Authors:** Kathleen P. Conte, Alison Laycock, Jodie Bailie, Veronica Matthews, Ross Bailie

**Affiliations:** 1 Health Systems, Management and Policy, Oregon Health Sciences- Portland State University School of Public Health, Portland State University, 1810 5th Ave, Suite 523V, Portland, OR 97201 USA; 2 University Centre for Rural Health, University of Sydney, Lismore, Australia; 3 Centre for Disability Research and Policy, University of Sydney, Camperdown, Australia; 4 School of Public Health, University of Sydney, Sydney, Australia

**Keywords:** Knowledge translation, Co-production, Collaboration, Knowledge mobilization, Systems thinking, Participatory research, Indigenous health, Continuous quality improvement

## Abstract

**Background:**

Effective knowledge translation ensures health care research has desired impacts – this is particularly important for Indigenous communities who have historically not benefited from research about and on them. Yet much knowledge translation in Indigenous contexts continues without community partnerships and disregards Indigenous values, languages and knowledge-sharing practices. Visual approaches can be engaging knowledge translation strategies that align with Indigenous knowledge translation traditions and amplify Indigenous perspectives. In this paper, we introduce a new tool we have coined a “Visual Bibliography” for knowledge generation and translation, developed within a large-scale, participatory research collaboration in Aboriginal and Torres Strait Islander health services.

**Methods:**

This case study explores the collaborative invention and development of the Visual Bibliography. Through a participatory process with Aboriginal and Torres Strait Islander and non-Indigenous members of the research collaboration, we synthesized and analyzed 92 research outputs – e.g., academic publications, reports, policy briefs – published by our collaboration focused on quality improvement in Aboriginal and Torres Straits Islander primary health care. Findings informed conceptual metaphors, infographics and other imagery that we combined into a single document that serves as a reference to all research outputs and communicates the values and history underpinning our collaboration.

**Results:**

Analysis and artistic experimentation with deep consideration of representation were combined to create the Visual Bibliography. Our process carefully balanced scientific accuracy with engaging depictions to convey complex, intersecting ideas which both communicate knowledge and generate new insights into health services research. The process itself fostered integrative knowledge translation and enabled participants to locate their contributions within a broader system of knowledge production.

**Conclusion:**

We believe the Visual Bibliography has broad potential within and beyond Indigenous knowledge translation contexts. It provides a tool for participatory co-creation, especially as part of an overarching embedded program of knowledge translation that can be responsive to Indigenous (and non-Indigenous) communities’ preferences for knowledge mobilization. By communicating complexity meaningfully and engagingly, it helps address a significant gap in knowledge translation.

**Supplementary Information:**

The online version contains supplementary material available at 10.1186/s12913-025-13936-7.

## Background

Health services research that results in benefits for Indigenous communities happens when Indigenous community members lead that research, or, are actively involved in its creation [1]. Research led by and with Indigenous communities facilitates the selection of research questions and priorities meaningful to that community, protocols that better uphold their local values, culture, and strengths, and thus, produces knowledge that is better translated into meaningful practice [2]. Despite this, Indigenous health continues to be produced without partnerships with communities [3], and within in Euro-Western paradigms that disregard or de-emphasize Indigenous values, languages and knowledge-sharing practices [1].

Knowledge translation is essential in ensuring research is converted into action and benefit. Knowledge translation is a complex process in which ideas, learnings, and ways of knowing are generated, shared, interpreted and applied throughout research endeavors [4]. From initial conceptualization to end-of-project outputs to actions and impacts that extend beyond the life of a single project, knowledge translation is most successful when it is embedded into the entire research lifecycle and is an active component of producing research. In Australia, knowledge translation is considered a foundation of ethical research with Aboriginal and Torres Strait Islander communities as articulated in many research guidelines [5, 6]. Knowledge translation that is meaningful, accurate and reported back to communities involved in the research helps ensure that research is beneficial to those communities. It also attends to a legacy of research done to Indigenous communities that is and was exploitative, taken without respect or care for their wellbeing or benefit [1, 7].

Effective knowledge translation for Aboriginal and Torres Strait Islander communities, as defined by Aboriginal and Torres Strait Islander peoples, includes that knowledge translation must be relevant to community needs and ways of mobilizing knowledge, embedded in research to address community priorities, and planned and evaluated in ways that reflect communities’ definitions of success [2]. Traditional research knowledge translation approaches – academic papers, conference presentations, etc. – often fail to meet Aboriginal and Torres Strait Islander communities’ needs and ways of knowledge sharing, as well as the needs of Indigenous communities’ needs more broadly. Instead, mechanisms such as storytelling, art, and visual methods are often more effective, interesting and culturally responsive [1, 2, 8].

The use of visual elements constitutes some of the most engaging types of translation products. Visual tools can make complicated stories accessible, immersive, and provide rich detail. Visual communications are inviting, reinforce meaning and facilitate memorability [9]. Recent research in Indigenous and Aboriginal and Torres Strait Islander knowledge translation underscores the value, desirability and effectiveness of visual knowledge translation products, e.g., visual storytelling, art, photos, infographics [2, 3]. Visual forms of communication developed with leadership or collaboration from Indigenous peoples may be a good fit for the communication needs and preferences of some communities versus traditional research outputs. Ultimately, effective and respectful knowledge translation with Indigenous communities is rooted in respectful relationships that uphold community rights, and adopting a broader scope of research translation tools is one of several needed approaches.

Our purpose in this paper is to introduce an innovative visual tool for knowledge generation and translation that we developed in the context of a large-scale research collaboration involving Aboriginal and Torres Strait Islander health services. The tool is called a “Visual Bibliography,” and was developed to facilitate research translation to a variety of audiences, including Aboriginal-controlled health organizations, community organizations and their representatives, and other researchers working in this space.

### Introducing visual bibliographies

The purpose of developing the “Visual Bibliography” concept was to summarize a broad, diverse body of evidence in an appealing and useful way. To our knowledge, we are the first to define the idea and coin the term “Visual Bibliography” in the peer-reviewed literature as searches on Google Scholar and PubMed yielded no sources. A few sources have used the language “visual bibliography” to refer to visual catalogues of art or books [10, 11] or as a synonym for bibliometric analyses that provide infographic-type summaries of meta-level data on a topic (i.e. number of sources, dates of publications, authors or nationalities represented, etc.) [12]. However, we go further to conceptualize a Visual Bibliography as a detailed, interactive teaching tool that provides deep insight into its topic – even possibly generating new insights through its creation. Like a bibliography, a Visual Bibliography is made up of collated sources on a specific topic. Like an annotated bibliography it also provides information on the content of those sources. The content, however, is highly curated, interpreted, and represented to produce an interactive and generative experience that supports knowledge translation.

Our development of a Visual Bibliography stemmed from our experience with the systems thinking tool called “rich picture,” a visual device used to depict a complex system [13]. A Visual Bibliography goes beyond simply depicting the content of a source (as an infographic might) to tell a complex story or stories. It aims to embrace complexity rather than simplifying information. Therefore, it has the potential for many applications: from providing an introduction to a complex topic to knowledge translation. A Visual Bibliography does this by acting as a map in that it provides a big-picture perspective of a topic that is made up of smaller, specific, detailed pieces of information. It uses visual techniques to communicate complex ideas that will be applicable for translating the complexities of research into practice. It is a wayfinding tool that helps orient a viewer to a particular topic area. There are multiple points of entry in which viewers can interact with and apply information in myriad ways, so there is no single way to approach reading or using it. This allows it to have multiple benefits as a knowledge translation tool, and be useful for different audiences and purposes.

In Table 1, we provide an overview of steps and considerations for creating a Visual Bibliography. We reference the table throughout the remainder of the paper as we describe our methods and results, offering insights, explanations, and examples for others interested in creating a Visual Bibliography for their own contexts.

**Table 1:** Steps and advice for creating a visual bibliography

Step	Description
Step 1: Identify the overarching purpose of the Visual Bibliography and the values underlying the work	Consider the purpose of the Visual Bibliography. It might be to provide an introductory overview of a complex topic, to provide insights into a methodological approach or, as in our case, to communicate the broad scope of research outputs developed by a large-scale collaboration. No matter the purpose, the positionality and intentions of the people involved in developing the Visual Bibliography will inform the choices made in generating it. Therefore, including a variety of perspectives - particularly desired end-users - will facilitate access and relevance of the final product.
Step 2: Define boundaries and identify sources of evidence	Define what will be included and what will not. A Visual Bibliography might be used alongside a traditional, systematic literature review as a complementary way to depict findings. But sources need not be limited to published research. Consider combining traditional and non-traditional research outputs such as policy briefs, fact sheets, artworks, storytelling, and other forms of evidence. It may also incorporate various types of knowledge including lay-knowledge and experiential knowledge through engaging in collaborative co-design.
Step 3: Review sources	Developing the imagery and metaphors for the Visual Bibliography (described in steps 4 & 5) requires strong knowledge of the included sources. The deeper the understanding, the greater the complexity that can be represented. In some cases, a structured review and synthesis of the sources to identify relevant elements such as topics, key findings, etc. may be sufficient. In other cases, in-depth analyses incorporating qualitative analytical approaches that align with the values identified in step 1 may be useful. Stakeholders central to the program of research (i.e., administrators, program directors, advisory committees) may be well-suited to this task given their embeddedness in the work.
Step 4: Develop an organizing schema	Sort, group and categorize sources. Experiment with various organizing schematics (e.g., by their key messages, purposes, or impacts). Visual imagery allows for the use of multiple schematics to simultaneously depict multi-dimensional aspects of a source. For example, icons or colors explained in legends could be used to depict type, topic, date of publication or authorship qualities (e.g., authorship involving students, community or Indigenous authors). Placement of items on the “map” can be used to depict relationships between items such as chronological development of ideas or impacts of research over time, conflicting or supporting findings, or ideas for future research. Organizing approaches might include by topic, key findings or messages, methodology, date of publication, output type, or others.
Step 5: Develop metaphors and/or stories	While developing organizing structures, consider what overarching messages or story(ies) are communicated by the body of work as a whole. These stories may be incorporated into the Visual Bibliography as sign points or tools that can help orient the viewer to central aspects of the body of work.
Step 6: Develop the Visual Bibliography “map” prototype	As underlying messages and organizing structures are developed, elements of the map - including both individual research outputs and overarching messages - can be physically placed in relation to each other. The placement of outputs can be used to tell stories within stories - e.g., items might be grouped by topic within an overarching key message. Experiment with how to visually represent both the research outputs and the overarching messages and stories and how these might be made to speak to each other. Consider various visualization techniques such as using graphs, icons, cartoons, colors, layering and labeling to communicate ideas. Develop a prototype of the visual bibliography via sketches on paper or whiteboards, or via computer applications (like PowerPoint).
Step 7: Test the prototype	Before finalizing the Visual Bibliography imagery, trial the prototype with various stakeholder groups. Seek feedback on initial impressions and interpretations to ensure intended meanings are being communicated and discuss what imagery might best communicate ideas. In our experience, an “unpolished” version invites more engagement and constructive criticism as people may hesitate to criticize versions that feel too complete.
Step 8: Develop or commission artwork	While some artists may be able to help develop ideas for visualization, it is important to enter this stage with a clear description of the key messages and metaphors and how they might be visualized. Be prepared for several rounds of revisions as meanings can dramatically shift as metaphors are re-interpreted into visual formats. Whenever possible, hire local artists and/or people with lived experience to develop the map or discrete elements that could be incorporated. If financial resources are unavailable, a simple Visual Bibliography may be developed using computer applications such as PowerPoint and/or clipart. Involve stakeholders in rounds of review and revision.
Step 9: Finalize the Visual Bibliography	Include a description and/or a legend to help introduce the viewer to the Visual Bibliography and how to use it. Assign identifiers to each source as it is depicted in the map. Provide a source list to accompany the identifiers used in the Visual Bibliography, or use hyperlinks in electronic documents to provide users direct access to sources.
Step 10: Disseminate and Evaluate	Dissemination of the final product in keeping with its purpose. It may be available as a print copy, or through online mediums. Presenting the Visual Bibliography as part of presentations to communities or researchers will help introduce the concept to audiences and provide an example of how it can be used in practice. Evaluation is an important component - seeking information on engagement, use of the tool, effectiveness and value will help build evidence about whether and in what contexts this tool is an effective knowledge translation approach.

## Methods

### Step 1. Identify the overarching purpose of the visual bibliography and the values underlying the work: our context

This case study describes the collaborative development and creation of a Visual Bibliography to facilitate knowledge translation. As outlined in Table 1, the first step is to clarify the purpose and values underlying the work to be represented in the Visual Bibliography, including whose voices will contribute to its creation. Our initiative occurred within a long-standing research collaboration in Aboriginal and Torres Strait Islander health services research. The Centre for Research Excellence in Integrated Quality Improvement (CRE-IQI) (2015–2019), was funded by the National Health and Medical Research Council of Australia. The CRE-IQI’s purpose was to improve Aboriginal and Torres Strait Islander health outcomes through research supporting continuous quality improvement (CQI) initiatives aimed at strengthening system-wide primary health care [14].

The collaboration consisted of Aboriginal and Torres Strait Islander and non-Indigenous members (hereafter referred to collectively as ‘CRE members’ or ‘members’) representing multiple levels of the health system, including researchers, policy officers, health service providers and practitioners and community members. CRE-IQI was co-governed by Aboriginal and Torres Strait Islander researchers and practitioners, and non-Indigenous researchers and staff. The governance and operation of the CRE-IQI strongly focused on collaboration, coproduction and power shifting from non-Indigenous to Indigenous research leadership, as reflected in project leadership arrangements, authorship and participation in CRE-IQI events over the five-year grant period. Governance committees oversaw everyday operations, strategic directions, and an overarching evaluation plan through which the Visual Bibliography work took place [15, 16]. As such, the creation of the Visual Bibliography was responsive to needs the governance committees identified as priorities for the overall CRE and its members. The team that then conceptualized, developed, and created the Visual Bibliography included both Aboriginal and non-Indigenous CRE-IQI members, and their contributions and roles are detailed in the authors’ information section (at the end of this paper). The development process was participatory and included the broader CRE membership at multiple points; these processes will be further described throughout the methods.

The CRE-IQI has published numerous papers detailing our core principles and approaches to collaborative work [17, 18] and evaluating the effectiveness and lessons from these approaches [16, 19, 20]. Integrated knowledge translation was a cross-cutting theme of the CRE-IQI and significant resources, including a dedicated position, were dedicated to its implementation across all work streams. Multiple types of knowledge translation products were developed across the life of the CRE to facilitate translation and uptake of research findings from 6 flagship projects, 12 aligned priority projects, and other research activities including numerous interactive workshops, a center-wide evaluation, and other complementing projects. At the time the final report was published in 2020, the products included 92 peer-reviewed publications, 7 policy/parliamentary submissions, 26 newsletters, 27 technical reports, 81 conference presentations, and 2600 responses received through an interactive data dissemination process to identify gaps and priorities to improve care [19].

### Step 1, continued: our research purpose

Most publication and dissemination outputs reported content from the individual CRE-IQI projects in which they were generated. A few cross-project research outputs were generated via the overarching evaluation and some policy documents; however, it was considered important to communicate the breadth of the work produced by the CRE and how the individual projects related to one another – preferably through a single resource where audiences could learn about and easily access the entire suite of CRE research outputs.

An important benefit of producing research within the CRE was the supported collaboration among a diverse network of over 85 contributing organizations who met at CRE-hosted biannual meetings and networking events. These activities facilitated active research translation among the network, resulting in knowledge sharing that facilitated idea spread, advancement and uptake of new research collaborations. Although peer-reviewed research outputs generally addressed specific research aims in line with clearly defined projects, in practice, knowledge was actively diffusing across projects, informing research approaches and advancements across the CRE and contributing to a broader body of work. As we’ve written elsewhere, “the knowledge generated by the CRE-IQI was not solely contained in the written publications, but was created, embodied and enacted among the members of the collaboration as they worked together to produce research” [21]. We wanted our knowledge translation resource to demonstrate this knowledge as a system, as more than the sum of the parts, produced through our values and ways of working.

Our purpose in creating a “Visual Bibliography,” therefore, was to provide a single resource with all research outputs from the CRE to educate users about the breadth of our research and impact. Additionally, it was important to our collaboration that we communicate the values guiding our work including working in partnership, respecting the past and present experiences of Indigenous people, ensuring Indigenous leadership and others [15]. Enduring partnerships are another guiding value and unique aspect of our work that we wanted to depict and helped guide our decision to reflect our work using imagery.

### Step 2. Define boundaries and identify sources of evidence: our focus

Our interest in developing a Visual Bibliography was to compile and communicate information about a complex and diverse work program that consisted of multiple projects. Therefore, the scope – or “boundary” – of our Visual Bibliography was broadly defined (see Step 2 in Table 1) to include all projects authors self-identified as being part of CRE-IQI (even if they were not funded by CRE-IQI). Given the breadth of our products, however, we limited our sources to the 92 peer-reviewed publications, but complemented this academic knowledge with lay-knowledge in a participatory process (described below).

### Step 3. Review sources: our analysis process

While there are many ways to conduct a synthesis and analysis of content for the Visual Bibliography, we combined several approaches to analyzing our sources. As described in Step 3, the greater the understanding, the greater the complexity that can be represented. We undertook an in-depth synthesis and analysis of all peer-reviewed publications from the CRE-IQI published between 2014 and 2019. We developed a co-produced synthesis process in which a team of Aboriginal and Torres Strait Islander and non-Indigenous CRE members conducted a structured review and synthesis of these publications. The synthesis was then used in a participatory workshop with 23 CRE members who conducted further analysis to organize research and generate overarching findings statements for our final report. The methods and findings of the structured and participatory synthesis are reported in detail elsewhere [21].

In brief, first a small group of CRE members undertook a review and synthesis of all the published research outputs of the CRE-IQI (*n* = 77). The group, comprising Aboriginal and Torres Strait Islander and non-Indigenous researchers, captured information from sources about topics, CRE-IQI aims addressed by the sources, key messages and findings, source type, and others. Subsequently, a participatory synthesis workshop involved 33 CRE members in reviewing, discussing and generating key messages for dissemination, published in a final report [19].

### Steps 4–8. Developing the visual bibliography: iterating ideas, metaphors, and visuals

Although several steps in Table 1 are iterative, the development of the visuals is especially so. Developing the visuals for the bibliography involves multiple steps including developing an organizing schema, interpreting these schemas as metaphors and stories, prototyping the map, developing the artwork, and seeking feedback with alternative interpretations. We briefly introduce our process here, provide more guidance in Table 1, and describe our outcomes in greater detail in the Results.

We collectively analyzed synthesis data for key messages, values guiding decisions and priorities/recommendations for next steps, and kept notes and sketches of overarching themes and ideas that extended across sources. The data collected and generated from the synthesis process was used to develop the Visual Bibliography. Aboriginal and Torres Strait Islander people were involved at all points in the development of the Visual Bibliography, including in developing and publishing many of the sources, conducting the initial literature syntheses and participatory review, providing feedback on the Visual Bibliography and in the authorship of this paper (see author information).

**Fig. 1:**
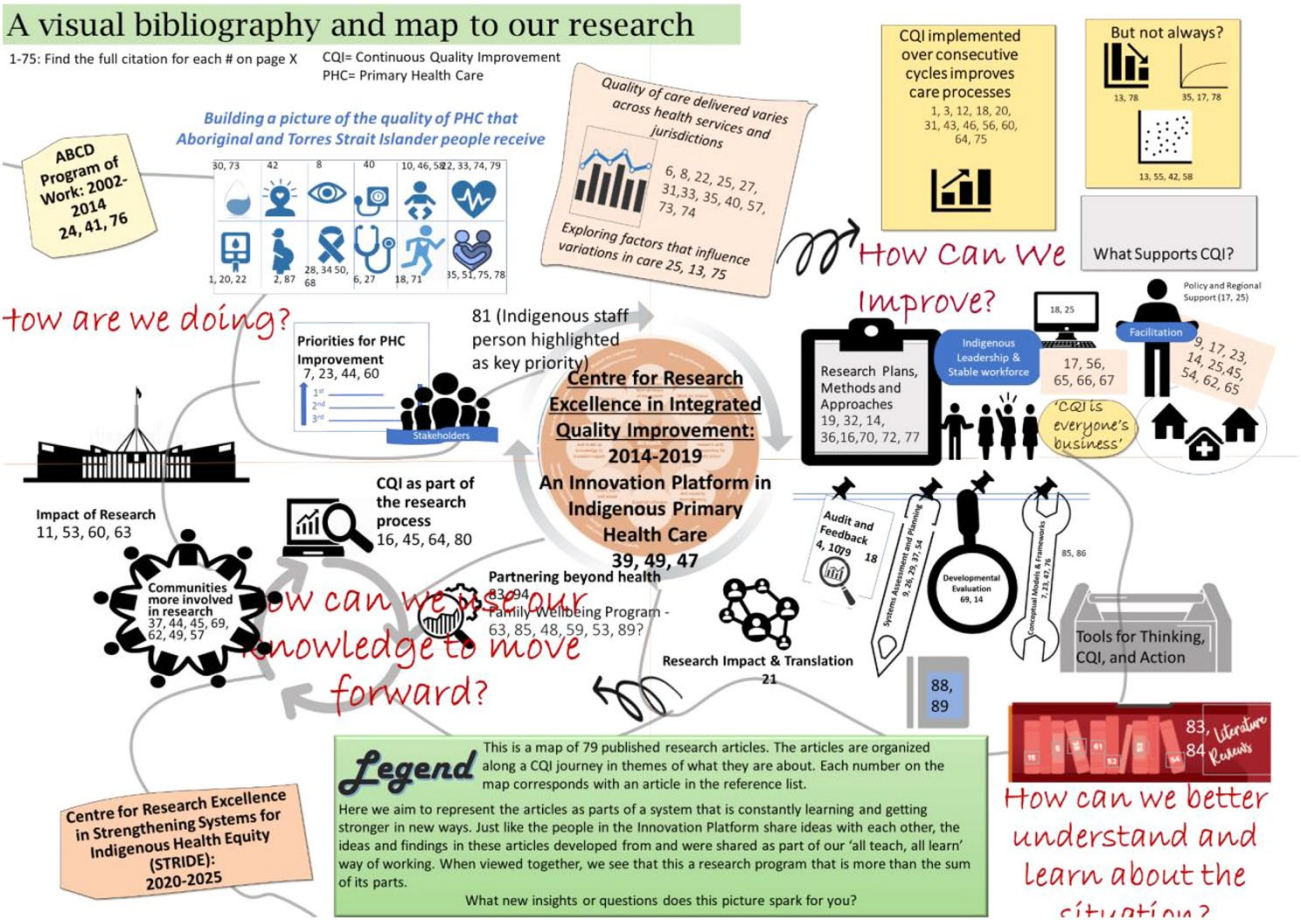
Draft rendering of the visual bibliography developed in PowerPoint, for presentation to collaboration participants for feedback

After developing various sketches, we used PowerPoint to develop an initial prototype, with clipart icons as temporary placeholders that could be disseminated for feedback (See Fig. 1). This draft was presented in a workshop to the CRE-IQI at a biannual meeting in late 2019 for comment and feedback. Fifty-five members reviewed the draft and provided critical feedback on resonant and problematic metaphors in an interactive workshop. Participants viewed the prototype, identified areas of confusion and offered interpretations of the depictions used that were helpful in assessing whether the imagery and metaphors were problematic or communicating the messages we intended. Those who provided written feedback following the workshop, and who are quoted in this paper, gave written permission for their quotes to be used.

After we felt confident that the metaphors and imagery we selected were a good fit for the story we wished to communicate, we employed a graphic artist to develop the finalized product. We provided the draft we created and developed a design brief that provided a detailed description of the content and metaphors we wanted to visually represent. Multiple iterations of design and review were undertaken before the final product.

### Steps 9–10. Finalizing, disseminating and evaluating

Our final Visual Bibliography is available in a printable version where a list of publications that correspond to the numbers is included on the back. It is also offered as a web-accessed or downloadable e-file where clicking on the number will directly link users to the online publication record [19]. The bibliography was published and disseminated as part of our final report and sent to a range of users including researchers, Aboriginal community-controlled health organizations, policy makers, and community members. Peer-review articles and other research outputs continued to be published during and after the synthesis processes were undertaken. Because we wanted the Visual Bibliography to be as comprehensive as possible, we incorporated newer publications into the design by mapping them to the organizing themes developed through the process described above. The final Visual Bibliography included a total of 92 sources, including 90 peer-reviewed publications and 2 books.

We sought informal feedback on the Visual Bibliography by asking research partners and CRE-IQI participants and investigators about how they have used the tool since its publication. However, a formal evaluation of the Visual Bibliography was beyond the scope of this project.

## Results

### Presenting our visual bibliography

**Fig. 2:**
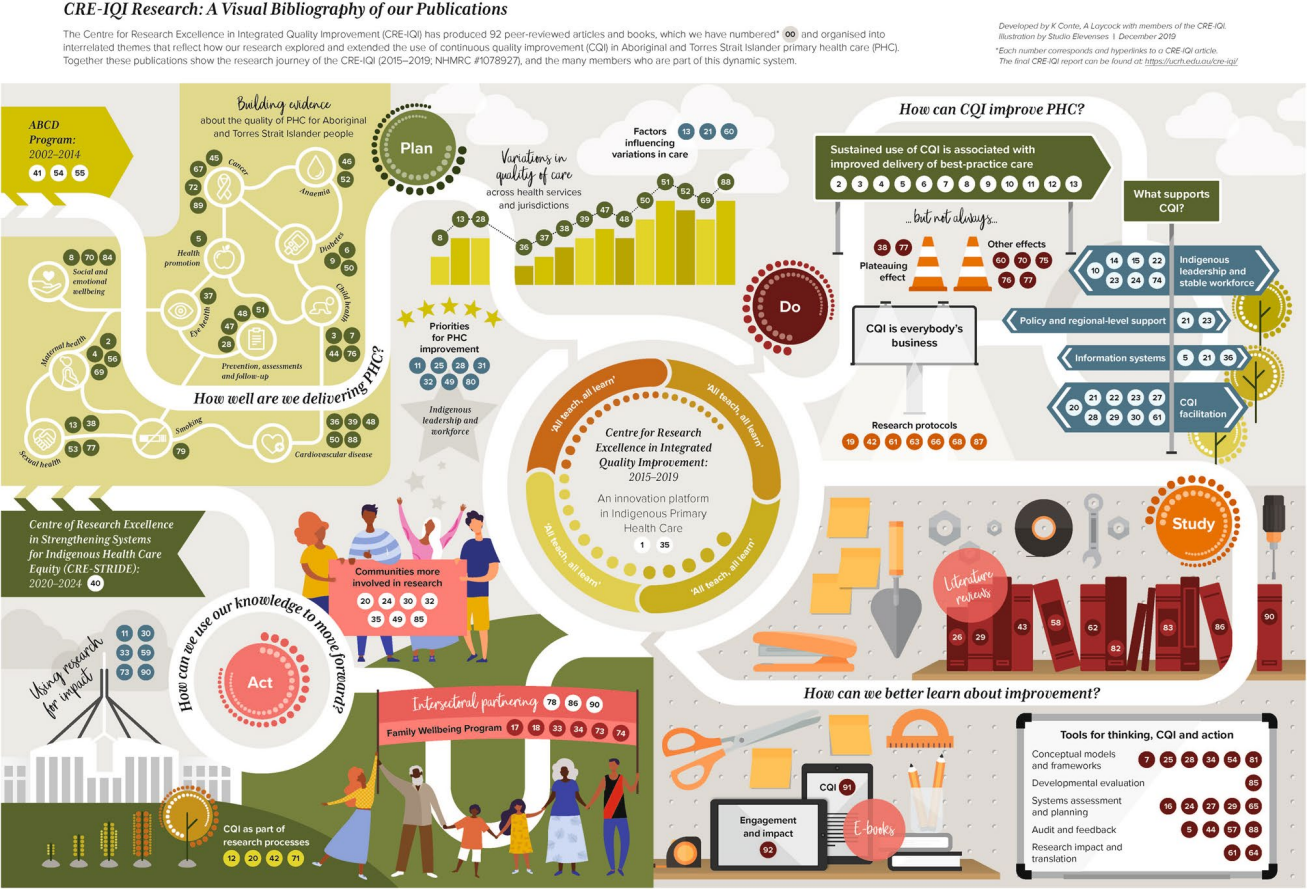
Visual bibliography of publications produced by the centre for research excellence in integrated quality improvement

**Fig. 3:**
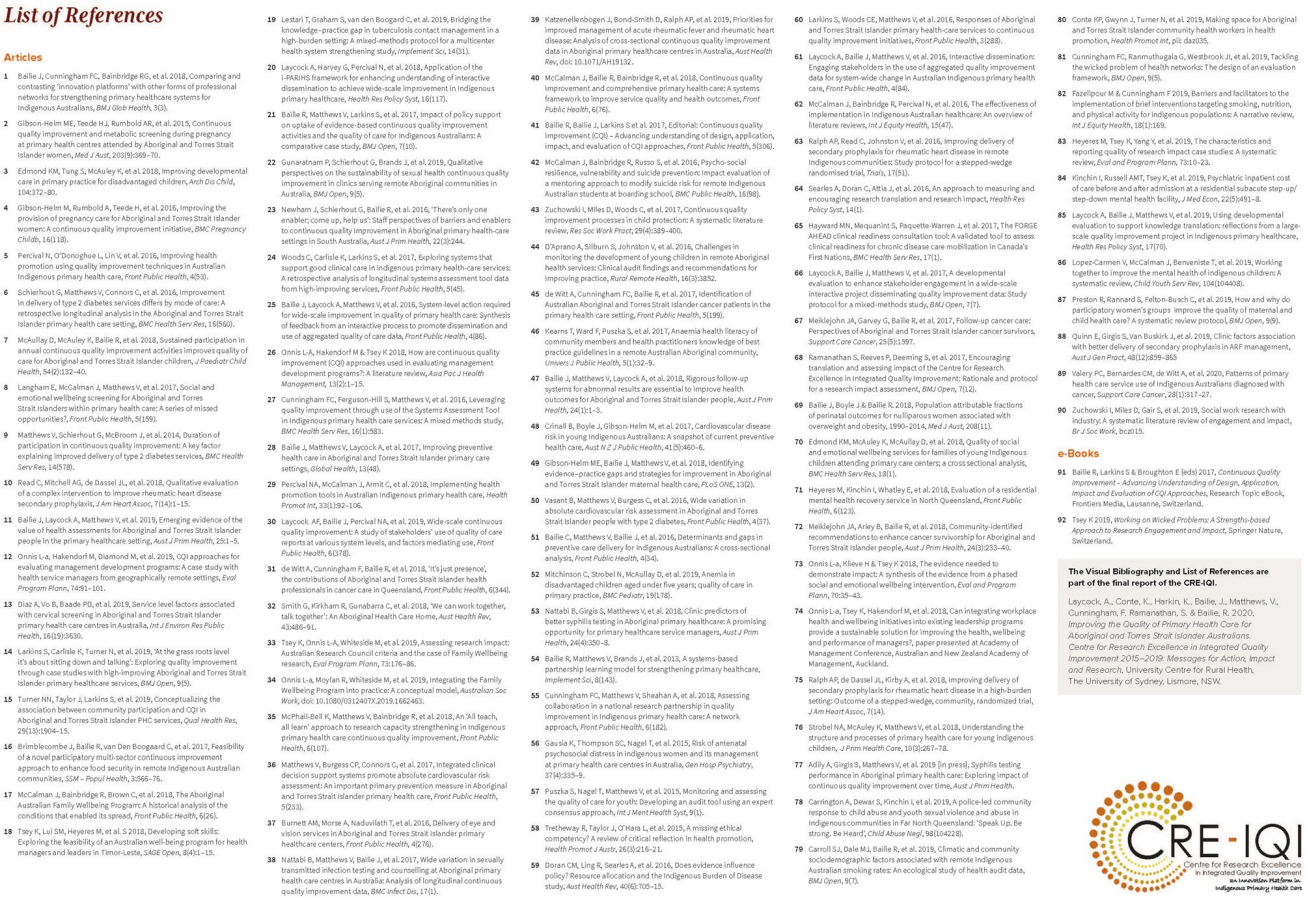
Visual bibliography reference list. Each number on the visual bibliography is linked to a research output which can be found on the reference list printed on the back, or by clicking on a hyperlink in the PDF version

The Visual Bibliography of the CRE-IQI is presented in Figures 2 and 3 (An interactive, to scale version of the Visual Bibliography is presented in Supplementary Figure 1). The map depicts an overall story, or journey, of the CRE-IQI including the collaboration grants that preceded and succeeded it. The main contents are the citations of the 92 CRE-IQI research products. Like a bibliography, each citation is numbered and its corresponding citations can be found in a reference list. Additionally, each number is hyperlinked directly to the online publication. The citations are organized by interrelated themes and subthemes that reflect the overall focus of the CRE-IQI, e.g., CQI research aimed at health system improvement. The main themes include:

1. how well (or not) primary health care is delivered for Aboriginal and Torres Strait Islander peoples on a range of health topics, including cancer care, child health care, cardiovascular disease care, and more;
2. research documenting and evaluating how CQI has been applied to improve primary health care services and illustrates key findings about what factors support successful CQI (i.e., Indigenous leadership, facilitation, information systems, and supportive contexts);
3. how to learn about or study improvement including conceptual models and frameworks developed within the collaboration, developmental evaluation, and tools for conducting CQI; and
4. using knowledge to move forward including articles that apply knowledge for action and impact, research about community involvement in research, intersectoral partnerships, and impacting policy and practice.

It is intended that users can interact with the map to learn about the overall themes addressed by CRE-IQI research, learn key influential findings that extend across projects, and simultaneously find and easily locate specific articles on topics of interest within those themes. For example, a user interested in quality improvement may learn about how ongoing CQI efforts result in improvement in health outcomes, and then locate articles that detail specific evidence-based facilitators. Or they may come to the Visual Bibliography looking for a citation on maternal health care statistics for Aboriginal and Torres Strait Islander peoples, and likewise find linkages to articles on infant and child health. Additionally, the map is also meant to provide an overview of the CRE’s work over time and ways of working.

In the next sections, we describe findings and learnings from our process of developing the Visual Bibliography, providing more insights behind the guidance outlined in Table 1.

### Defining the purpose and scope of work (Steps 1–3)

We found that developing the imagery and metaphors for the Visual Bibliography was enhanced by our deep knowledge of the sources. Through the in-depth synthesis process described above, our team became deeply knowledgeable about the content, findings, and methods used for all sources. We were able to draw on this knowledge to inform the organization and visual connections between sources, and to develop teaching metaphors.

Although we tried to include all research outputs, we were still challenged to establish boundaries for the Visual Bibliography. As previously mentioned, our work was undertaken in a network of researchers whose work benefited from and was informed by the integrated knowledge translation program. Their work may have been informed by the CRE-IQI, but may not have been specifically tagged as “affiliated” with the CRE. We choose to omit some research outputs that may be of value such as policy briefs, webpages, toolkits, and others. As described above, research outputs continued to be published during the development of the visual. As much as possible, we tried to include all research publications available at the time of developing the publishing the Visual Bibliography, but publications occurred after its completion and are still forthcoming at the time of (and including) this manuscript. Therefore, even this large, comprehensive work was in some ways incomplete.

### Developing the visuals (Steps 4–6)

Developing the visuals, including an organizing schema, metaphors and developing main themes required both artistic and scientific processes. It was artistic in that we sketched, colored, imagined, brainstormed and otherwise trialed many different visual renderings to explore the many options through which we could present the information. It was scientific in that it was a systematic process of reviewing, documenting, and re-reviewing articles to ensure their main findings were accurately represented and interpreted in the metaphors used. In an iterative fashion, we reviewed sources, trialed various organizing schemas, stories and visualizations to identify and depict the main themes. We found that we needed to experiment with a variety of visualizations that might help us best display all the research outputs in relation and conversation to one another in a visual format.

Given the focus of the CRE’s work on quality improvement, a CQI cycle [22] – “plan, do, study, act” – was determined to be a useful overall organizing schema depicting both the kinds of research we produced and our overall commitment to working as a learning system. Therefore, each quadrant of the bibliography corresponds with a step of the cycle. The main themes and subthemes, as described above, are organized within this overarching schema. In addition to depicting sources linked to each subtheme, we also incorporated different visual techniques to communicate more detailed information about the sources. For example, we used icons to depict topic areas of substantial baseline research on a range of health issues; hardware tools and books symbolized CQI tools and resources developed by the collaborative; and cartoon-like images to represent people in a neutral, but representative way.

Zooming out, the Visual Bibliography also encompasses a pathway to denote the long-standing work of the collaboration that preceded the CRE-IQI and provided the foundation for the trusting relationships on which the CRE-IQI was built; it was important to CRE members to represent these features [17]. The pathway travels through each quadrant whilst encircling the CRE-IQI logo in the center, and is surrounded by its guiding motto: “All teach, All learn” [18]. This motto elevates the value we place on mutual learning. Finally, the path continues into the future reflecting the next iteration of the collaboration in the Aboriginal-led Centre for Research Excellence: Strengthening Systems for Indigenous Health care equity (CRE-STRIDE) 2020–2024. The color scheme, central icon, and other design elements are drawn from CRE-IQI branding used in logos and reports.

We found that visual imagery allowed for the use of multiple, overlaying schematics, while at the same time enabling a product that could be read without a legend and accompanied by only a brief description at the top. The placement of outputs could be used to tell stories within stories, e.g., items grouped by topic within an overarching key message. We grappled with the placement of items in relationship to one another, especially because items often could fit into more than one category. We placed articles with conflicting or supporting findings near each other, and used the pathway metaphor to illustrate ideas for future research.

### Prototyping and finalizing the design (Steps 7–9)

Overall, feedback from CRE-IQI members during the interactive workshops was positive. Edits suggested by attendees involved making the overall feel and aesthetic better represent Aboriginal and Torres Strait Islander people and design. There were also edits to images and wording that more accurately reflect the research findings, and specific recommendations about what content to prioritize. For example, a strong research outcome across our program of work was evidence that sustained use of CQI is associated with improved delivery of best-practice care. Twelve articles supported this statement, but two articles found that CQI improvement plateaued over time and five articles showed ambiguous effects. We originally presented these articles together in a generalized category outlining variations in the impact of CQI on practice (as shown in Fig. 1). Yet our members agreed that the evidence supporting sustained CQI delivery was strong, and so we chose to emphasize this message while less prominently acknowledging variations found among a small number of scientific studies (as shown in Fig. 2). Similarly, participants recommended highlighting enabling factors for CQI by naming them in the visual rather than aggregating them under an overarching heading.

### Dissemination and feedback (Step 10)

Overall, we received positive feedback from partner organizations who were sent the tool as part of our dissemination. Additionally, we have heard from external organizations who learned of the tool through CRE-IQI members. Several users described posting the resource in their office as a tool to quickly find needed citations. In addition, the idea has been taken up by other organizations as a tool to communicate their complex data. For example, a partnering Aboriginal-controlled community health organization emailed us the following feedback:

*The concept of a continuous visual (or lifelong) story*,* as depicted in the Visual Bibliography*,* is appealing to us as a way of representing our five-year compilation of child health data … We notice that those that take the time to review [our] data report seem to concentrate on singular items where they*,* their team*,* or organisation have done well. We need to put the pieces together in a way that our staff and our partners from government and non-government agencies can see the entire picture and how data are interrelated… With our next five-year data report*,* we will draw on the Visual Bibliography idea to create a visual story at the same time*,* and release both publications simultaneously.*

The organization found value in the Visual Bibliography approach as a tool to present research findings as a comprehensive picture that can both celebrate successes and facilitate discussion on areas of improvement.

The evaluation team from another Centre of Research Excellence described its value in communicating complexity. They said:

*The Visual Bibliography is an engaging document that provided us with inspiration for how to approach our own Centre for Research Excellence evaluation. We regarded the Visual Bibliography as a tool to frame complex and inter-related research outputs in a clear and easily understood format that could demonstrate the value and meaning of the research in context. … We have adopted a similar thematic process to frame our research impact.*

Finally, members of the CRE-IQI themselves described how useful it was to be involved in the process of making the Visual Bibliography. As previously discussed, the CRE was made up of many researchers and organizations, each with their own specialty, focused projects, and remits. The goal of our integrated knowledge translation work was to facilitate connections among these various entities, demonstrating and facilitating linkages between research topics and activities. While we undertook various knowledge translation activities to this end, the participatory workshops to develop the Visual Bibliography also facilitated intra-CRE knowledge translation. As one CRE member said:

*Participating in the development of the CRE-IQI Visual Bibliography is one of the most useful research collaboration activities that I have been involved in… As a multi-disciplinary researcher*,* I often feel isolated because it is not always easy to see where my research fits within a specific discipline. I found that in developing the CRE-IQI Visual Bibliography it was clearer that my research not only was a good fit for the CRE-IQI*,* but the visualization also showed me exactly where it fits in relation to the other research within the CRE-IQI.*

## Discussion

The case of the “Visual Bibliography” holds promise as an innovative tool for facilitating knowledge translation in Indigenous health research contexts, and we believe it has application for broader use for other research groups that similarly produce complex and diverse research outputs.

Conducting a full evaluation of the Visual Bibliography’s effectiveness as a translation tool was beyond our scope, and we hope that by sharing this idea, future research and evaluation of this and other novel translation tools will be possible. Early feedback, however, demonstrates that other organizations find value in its ability to present research findings in a way that retains complexity. This is of particular importance because science communication guidelines emphasize simplified messages, focused on particular target groups [23, 24]; while clear and directed, this form of communication risks being reductionistic, reducing complexity instead of better enabling audiences to grapple with it. Emerging research suggests that engaging audiences in complexity may help people better understand the limits of science and increase credibility and trust of scientists [25]. Narratives have been frequently used as a tool to present complex issues, but concerns with narrative approaches is that it reduces audience engagement with numbers and statistical findings [26]. Visual bibliographies may attend to this issue by presenting both a narrative alongside numerical information interwoven into the storyline.

Another promising benefit of the Visual Bibliography is as a tool of *integrated* knowledge translation, i.e. facilitating active collaborative research between researchers and end-users [27]. The Visual Bibliography was a useful complement to our overall suite of knowledge translation activities by demonstrating our diverse work outputs as part of a broader system of research aimed at healthcare improvement. Thus, it helped Aboriginal and Torres Strait Islander and non-Indigenous members within our network see how their contributions are linked to and complement each other. Generating these insights may be fruitful for new collaborations and linkages that could attend to cross-disciplinary or cross-sector problems by enabling research partners to better understand how their work connects to systemic issues.

Creating the Visual Bibliography via a participatory process also facilitated these insights by engaging researchers and community partners, including both Aboriginal community members and Aboriginal people in service provider roles, in direct conversations about linkages between work programs and implications for the field. While it may be possible for one person or a small team to create a Visual Bibliography, we feel that its value is fully realized when part of a participatory approach; this theory is supported by research demonstrating the effectiveness of more active forms of knowledge translation over passive forms [28]. As previously described, participatory and co-production activities that are led by or with Aboriginal and Torres Strait Islander communities – including service providers and researchers – are core to respectful, effective, and meaningful co-production with those communities [1, 2, 29] and we believe our process illustrates this kind of successful co-production. Other studies of the CRE-IQI co-production and power-shifting models of research (in which the Visual Bibliography was undertaken) underscore the success and learnings of our approach [16, 20]. Further, participatory approaches to knowledge translation are increasingly recognized as a best practice approach for their ability to improve the value, acceptance and application of science in practice [30]. More research and innovation are needed, however, on methods that truly facilitate equal partnership between researchers and various audiences. We offer the Visual Bibliography tool as one approach that may help fill this gap.

Challenges we encountered in developing the Visual Bibliography centered on prototyping and trialing imagery. We found generating visuals of our findings could yield unintended, confusing or undesirable interpretations. A benefit of using visual imagery is that its ambiguity can allow audiences to better see themselves as part of the art, and to engage with and derive their own meanings. But this ambiguity could be perceived as a risk to science communication. Therefore, a balance needs to be struck between offering enough ambiguity in imagery that various audiences can find value in the Visual Bibliography as a useful tool for their varying purposes, while also ensuring clear communication around research findings to reduce misinterpretations. In our case, we addressed this problem by representing our research at a generally descriptive level coupled with clear statements about important findings.

Another challenge was that, as (mostly) non-artistic researchers, communicating our vision for an artistic rendering to an outside artist was a challenge. In our experience, graphic facilitators seem well-attuned to this task given their familiarity with depicting ideas as a system. In other projects we had engaged graphic facilitators earlier in the process by including them in workshops where they could be part of and become embedded in the context and meaning-making work. We did not have sufficient resources, however, to involve them earlier in this project. Therefore, it was important that we be as clear as possible about how the visual should look and have most of the visualization ideas already mapped out. Involving an artist earlier on may have reduced the amount of revisions we undertook. As such, without their early involvement it was important to devote resources to revising and seeking feedback to ensure key messages accurately were conveyed.

A final, and important, area for improvement is that this work is part of an ongoing process of shifting leadership to Aboriginal and Torres Strait Islander researchers in our context. While we worked in close partnership to develop, test, refine and disseminate the Visual Bibliography, ideally, future work will – and is being [31, 32] – led by Aboriginal peoples themselves. In other CRE-IQI projects we employed Aboriginal artists to create iconography that could be incorporated into visual products and to develop knowledge translation outputs [33], though that was out of scope for this project. Moving forward, we include line-items in our grants and contracts for hiring artists to develop knowledge translation. Further, the Visual Bibliography was developed for the wide range of stakeholder groups interested in our CQI research, including Aboriginal Community Controlled Health Organizations. It is a bibliography of a large suite of peer reviewed research papers, so our discussion and dissemination are inevitably researcher and system focused. We provide a tool that our members – including our Aboriginal and Torres Strait Islander members who are researchers, practitioners, and members of their own communities – can use to facilitate research translation to practice and policy that ultimately meets the needs and reflect the priorities of the specific communities they serve. While future research is needed to examine whether the Visual Bibliography effectively achieves this goal, we believe it meets a gap for translation tools that facilitate multi-level and intersectoral knowledge translation necessary for impact [34].

## Conclusions

We coin the term “Visual Bibliography” as a visual tool that provides a map to a diverse body of research, produced by a large collaboration in Aboriginal and Torres Strait Islander health care improvement over multiple years. Our intention here is to report on an innovative idea that other large-scale research collaboratives could adopt to disseminate a large body of diverse research, as well as a process for participatory and integrated knowledge translation with Aboriginal and Torres Strait Islander researchers and service providers who serve their local communities. The Visual Bibliography approach provides a compelling way to tell a story of a research collaboration while providing a resource to research outputs the collaboration produced. It acts as a tool for engagement both in the act of its participatory creation process, and for communicating complexity in a meaningful way that may be adapted to culturally-appropriate knowledge translation for Indigenous communities. We chose to tell a story about the breadth and depth of the new knowledge we created, our publications and outputs, our evolution over time, and our ways of working and values. Other collaborations may wish to tell about their impact, barriers surmounted, and achievements. Or Visual Bibliographies may be used to summarize literature on a complex topic and could be used to complement systematic literature reviews. We believe the Visual Bibliography has many potential applications and is a strategy worth sharing as another tool for knowledge translation.

## Electronic Supplementary Material

Below is the link to the electronic supplementary material.

Supplementary Figure 1

## Data Availability

All data generated or analyzed during this study are included in this or linked published articles.

## Abbreviations

CRE-IQI: Center for Research Excellence in Integrated Quality Improvement
CQI: Continuous Quality Improvement
