# Supplementary material for: Introducing “Visual bibliographies” as a novel tool for communicating complexity: a knowledge translation case study from Aboriginal and Torres Strait Islander primary health care research
Source: BMC Health Serv Res. 2026 Jan 23;26:254. doi: 10.1186/s12913-025-13936-7 (PMC12911360; doi:10.1186/s12913-025-13936-7)
Supplement: Supplementary file 1 — Supplementary Figure 1 [file 12913_2025_13936_MOESM1_ESM.pdf]

CRE-IQI Research: A Visual Bibliography of our Publications

The Centre for Research Excellence in Integrated Quality Improvement (CRE-IQI) has produced 92 peer-reviewed articles and books, which we have numbered* 00 and organised into interrelated themes that reflect how our research explored and extended the use of continuous quality improvement (CQI) in Aboriginal and Torres Strait Islander primary health care (PHC). Together these publications show the research journey of the CRE-IQI (2015–2019; NHMRC #1078927), and the many members who are part of this dynamic system.

Developed by K Conte, A Laycock with members of the CRE-IQI.
Illustration by Studio Elevenses | December 2019

*Each number corresponds and hyperlinks to a CRE-IQI article.
The final CRE-IQI report can be found at: https://ucrh.edu.au/cre-iqi/

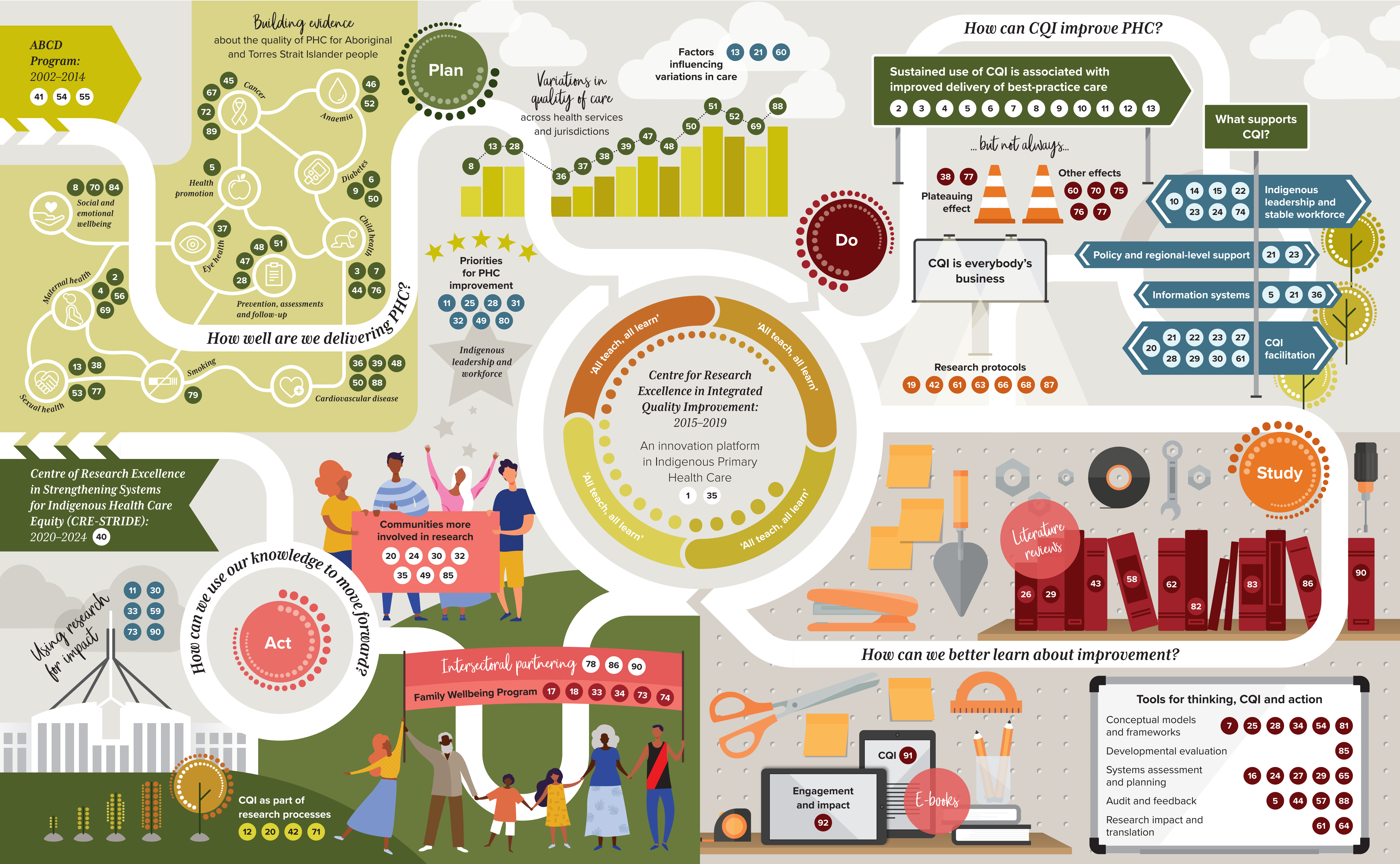

List of References

Articles

1 Bailie J, Cunningham FC, Bainbridge RG, et al. 2018, Comparing and contrasting 'innovation platforms' with other forms of professional networks for strengthening primary healthcare systems for Indigenous Australians, *BMJ Glob Health*, 3(3).

2 Gibson-Helm ME, Teede HJ, Rumbold AR, et al. 2015, Continuous quality improvement and metabolic screening during pregnancy at primary health centres attended by Aboriginal and Torres Strait Islander women, *Med J Aust*, 203(9):369–70.

3 Edmond KM, Tung S, McAuley K, et al. 2018, Improving developmental care in primary practice for disadvantaged children, *Arch Dis Child*, 104:372–80.

4 Gibson-Helm M, Rumbold A, Teede H, et al. 2016, Improving the provision of pregnancy care for Aboriginal and Torres Strait Islander women: A continuous quality improvement initiative, *BMC Pregnancy Childb*, 16(118).

5 Percival N, O'Donoghue L, Lin V, et al. 2016, Improving health promotion using quality improvement techniques in Australian Indigenous primary health care, *Front Public Health*, 4(53).

6 Schierhout G, Matthews V, Connors C, et al. 2016, Improvement in delivery of type 2 diabetes services differs by mode of care: A retrospective longitudinal analysis in the Aboriginal and Torres Strait Islander primary health care setting, *BMC Health Serv Res*, 16(560).

7 McAullay D, McAuley K, Bailie R, et al. 2018, Sustained participation in annual continuous quality improvement activities improves quality of care for Aboriginal and Torres Strait Islander children, *J Paediatr Child Health*, 54(2):132–40.

8 Langham E, McCalman J, Matthews V, et al. 2017, Social and emotional wellbeing screening for Aboriginal and Torres Strait Islanders within primary health care: A series of missed opportunities?, *Front Public Health*, 5(159).

9 Matthews V, Schierhout G, McBroom J, et al. 2014, Duration of participation in continuous quality improvement: A key factor explaining improved delivery of type 2 diabetes services, *BMC Health Serv Res*, 14(578).

10 Read C, Mitchell AG, de Dassel JL, et al. 2018, Qualitative evaluation of a complex intervention to improve rheumatic heart disease secondary prophylaxis, *J Am Heart Assoc*, 7(14):1–15.

11 Bailie J, Laycock A, Matthews V, et al. 2019, Emerging evidence of the value of health assessments for Aboriginal and Torres Strait Islander people in the primary healthcare setting, *Aust J Prim Health*, 25:1–5.

12 Onnis L-a, Hakendorf M, Diamond M, et al. 2019, CQI approaches for evaluating management development programs: A case study with health service managers from geographically remote settings, *Eval Program Plann*, 74:91–101.

13 Diaz A, Vo B, Baade PD, et al. 2019, Service level factors associated with cervical screening in Aboriginal and Torres Strait Islander primary health care centres in Australia, *Int J Environ Res Public Health*, 16(19):3630.

14 Larkins S, Carlisle K, Turner N, et al. 2019, 'At the grass roots level it's about sitting down and talking': Exploring quality improvement through case studies with high-improving Aboriginal and Torres Strait Islander primary healthcare services, *BMJ Open*, 9(5).

15 Turner NN, Taylor J, Larkins S, et al. 2019, Conceptualizing the association between community participation and CQI in Aboriginal and Torres Strait Islander PHC services, *Qual Health Res*, 29(13):1904–15.

16 Brimblecombe J, Bailie R, van Den Boogaard C, et al. 2017, Feasibility of a novel participatory multi-sector continuous improvement approach to enhance food security in remote Indigenous Australian communities, *SSM – Popul Health*, 3:566–76.

17 McCalman J, Bainbridge R, Brown C, et al. 2018, The Aboriginal Australian Family Wellbeing Program: A historical analysis of the conditions that enabled its spread, *Front Public Health*, 6(26).

18 Tsey K, Lui SM, Heyeres M, et al. S 2018, Developing soft skills: Exploring the feasibility of an Australian well-being program for health managers and leaders in Timor-Leste, *SAGE Open*, 8(4):1–15.

19 Lestari T, Graham S, van den Boogard C, et al. 2019, Bridging the knowledge–practice gap in tuberculosis contact management in a high-burden setting: A mixed-methods protocol for a multicenter health system strengthening study, *Implement Sci*, 14(31).

20 Laycock A, Harvey G, Percival N, et al. 2018, Application of the i-PARIHS framework for enhancing understanding of interactive dissemination to achieve wide-scale improvement in Indigenous primary healthcare, *Health Res Policy Syst*, 16(117).

21 Bailie R, Matthews V, Larkins S, et al. 2017, Impact of policy support on uptake of evidence-based continuous quality improvement activities and the quality of care for Indigenous Australians: A comparative case study, *BMJ Open*, 7(10).

22 Gunaratnam P, Schierhout G, Brands J, et al. 2019, Qualitative perspectives on the sustainability of sexual health continuous quality improvement in clinics serving remote Aboriginal communities in Australia, *BMJ Open*, 9(5).

23 Newham J, Schierhout G, Bailie R, et al. 2016, 'There's only one enabler; come up, help us': Staff perspectives of barriers and enablers to continuous quality improvement in Aboriginal primary health-care settings in South Australia, *Aust J Prim Health*, 22(3):244.

24 Woods C, Carlisle K, Larkins S, et al. 2017, Exploring systems that support good clinical care in indigenous primary health-care services: A retrospective analysis of longitudinal systems assessment tool data from high-improving services, *Front Public Health*, 5(45).

25 Bailie J, Laycock A, Matthews V, et al. 2016, System-level action required for wide-scale improvement in quality of primary health care: Synthesis of feedback from an interactive process to promote dissemination and use of aggregated quality of care data, *Front Public Health*, 4(86).

26 Onnis L-A, Hakendorf M & Tsey K 2018, How are continuous quality improvement (CQI) approaches used in evaluating management development programs?: A literature review, *Asia Pac J Health Management*, 13(2):1–15.

27 Cunningham FC, Ferguson-Hill S, Matthews V, et al. 2016, Leveraging quality improvement through use of the Systems Assessment Tool in Indigenous primary health care services: A mixed methods study, *BMC Health Serv Res*, 16(1):583.

28 Bailie J, Matthews V, Laycock A, et al. 2017, Improving preventive health care in Aboriginal and Torres Strait Islander primary care settings, *Global Health*, 13(48).

29 Percival NA, McCalman J, Armit C, et al. 2018, Implementing health promotion tools in Australian Indigenous primary health care, *Health Promot Int*, 33(1):92–106.

30 Laycock AF, Bailie J, Percival NA, et al. 2019, Wide-scale continuous quality improvement: A study of stakeholders' use of quality of care reports at various system levels, and factors mediating use, *Front Public Health*, 6(378).

31 de Witt A, Cunningham F, Bailie R, et al. 2018, 'It's just presence', the contributions of Aboriginal and Torres Strait Islander health professionals in cancer care in Queensland, *Front Public Health*, 6(344).

32 Smith G, Kirkham R, Gunabarra C, et al. 2018, 'We can work together, talk together': An Aboriginal Health Care Home, *Aust Health Rev*, 43:486–91.

33 Tsey K, Onnis L-A, Whiteside M, et al. 2019, Assessing research impact: Australian Research Council criteria and the case of Family Wellbeing research, *Eval Program Plann*, 73:176–86.

34 Onnis L-a, Moylan R, Whiteside M, et al. 2019, Integrating the Family Wellbeing Program into practice: A conceptual model, *Australian Soc Work*, doi: 10.1080/0312407X.2019.1662463.

35 McPhail-Bell K, Matthews V, Bainbridge R, et al. 2018, An 'All teach, all learn' approach to research capacity strengthening in Indigenous primary health care continuous quality improvement, *Front Public Health*, 6(107).

36 Matthews V, Burgess CP, Connors C, et al. 2017, Integrated clinical decision support systems promote absolute cardiovascular risk assessment: An important primary prevention measure in Aboriginal and Torres Strait Islander primary health care, *Front Public Health*, 5(233).

37 Burnett AM, Morse A, Naduvilath T, et al. 2016, Delivery of eye and vision services in Aboriginal and Torres Strait Islander primary healthcare centers, *Front Public Health*, 4(276).

38 Nattabi B, Matthews V, Bailie J, et al. 2017, Wide variation in sexually transmitted infection testing and counselling at Aboriginal primary health care centres in Australia: Analysis of longitudinal continuous quality improvement data, *BMC Infect Dis*, 17(1).

39 Katzenellenbogen J, Bond-Smith D, Ralph AP, et al. 2019, Priorities for improved management of acute rheumatic fever and rheumatic heart disease: Analysis of cross-sectional continuous quality improvement data in Aboriginal primary healthcare centres in Australia, *Aust Health Rev*, doi: 10.1071/AH19132.

40 McCalman J, Bailie R, Bainbridge R, et al. 2018, Continuous quality improvement and comprehensive primary health care: A systems framework to improve service quality and health outcomes, *Front Public Health*, 6(76).

41 Bailie R, Bailie J, Larkins S et al. 2017, Editorial: Continuous quality improvement (CQI) – Advancing understanding of design, application, impact, and evaluation of CQI approaches, *Front Public Health*, 5(306).

42 McCalman J, Bainbridge R, Russo S, et al. 2016, Psycho-social resilience, vulnerability and suicide prevention: Impact evaluation of a mentoring approach to modify suicide risk for remote Indigenous Australian students at boarding school, *BMC Public Health*, 16(98).

43 Zuchowski I, Miles D, Woods C, et al. 2017, Continuous quality improvement processes in child protection: A systematic literature review, *Res Soc Work Pract*, 29(4):389–400.

44 D'Aprano A, Silburn S, Johnston V, et al. 2016, Challenges in monitoring the development of young children in remote Aboriginal health services: Clinical audit findings and recommendations for improving practice, *Rural Remote Health*, 16(3):3852.

45 de Witt A, Cunningham FC, Bailie R, et al. 2017, Identification of Australian Aboriginal and Torres Strait Islander cancer patients in the primary health care setting, *Front Public Health*, 5(199).

46 Kearns T, Ward F, Puszka S, et al. 2017, Anaemia health literacy of community members and health practitioners knowledge of best practice guidelines in a remote Australian Aboriginal community, *Univers J Public Health*, 5(1):32–9.

47 Bailie J, Matthews V, Laycock A, et al. 2018, Rigorous follow-up systems for abnormal results are essential to improve health outcomes for Aboriginal and Torres Strait Islander people, *Aust J Prim Health*, 24(1):1–3.

48 Crinall B, Boyle J, Gibson-Helm M, et al. 2017, Cardiovascular disease risk in young Indigenous Australians: A snapshot of current preventive health care, *Aust N Z J Public Health*, 41(5):460–6.

49 Gibson-Helm ME, Bailie J, Matthews V, et al. 2018, Identifying evidence–practice gaps and strategies for improvement in Aboriginal and Torres Strait Islander maternal health care, *PLoS ONE*, 13(2).

50 Vasant B, Matthews V, Burgess C, et al. 2016, Wide variation in absolute cardiovascular risk assessment in Aboriginal and Torres Strait Islander people with type 2 diabetes, *Front Public Health*, 4(37).

51 Bailie C, Matthews V, Bailie J, et al. 2016, Determinants and gaps in preventive care delivery for Indigenous Australians: A cross-sectional analysis, *Front Public Health*, 4(34).

52 Mitchinson C, Strobel N, McAullay D, et al. 2019, Anemia in disadvantaged children aged under five years; quality of care in primary practice, *BMC Pediatr*, 19(178).

53 Nattabi B, Girgis S, Matthews V, et al. 2018, Clinic predictors of better syphilis testing in Aboriginal primary healthcare: A promising opportunity for primary healthcare service managers, *Aust J Prim Health*, 24(4):350–8.

54 Bailie R, Matthews V, Brands J, et al. 2013, A systems-based partnership learning model for strengthening primary healthcare, *Implement Sci*, 8(143).

55 Cunningham FC, Matthews V, Sheahan A, et al. 2018, Assessing collaboration in a national research partnership in quality improvement in Indigenous primary health care: A network approach, *Front Public Health*, 6(182).

56 Gausia K, Thompson SC, Nagel T, et al. 2015, Risk of antenatal psychosocial distress in indigenous women and its management at primary health care centres in Australia, *Gen Hosp Psychiatry*, 37(4):335–9.

57 Puszka S, Nagel T, Matthews V, et al. 2015, Monitoring and assessing the quality of care for youth: Developing an audit tool using an expert consensus approach, *Int J Ment Health Syst*, 9(1).

58 Tretheway R, Taylor J, O'Hara L, et al. 2015, A missing ethical competency? A review of critical reflection in health promotion, *Health Promot J Austr*, 26(3):216–21.

59 Doran CM, Ling R, Searles A, et al. 2016, Does evidence influence policy? Resource allocation and the Indigenous Burden of Disease study, *Aust Health Rev*, 40(6):705–15.

60 Larkins S, Woods CE, Matthews V, et al. 2016, Responses of Aboriginal and Torres Strait Islander primary health-care services to continuous quality improvement initiatives, *Front Public Health*, 3(288).

61 Laycock A, Bailie J, Matthews V, et al. 2016, Interactive dissemination: Engaging stakeholders in the use of aggregated quality improvement data for system-wide change in Australian Indigenous primary health care, *Front Public Health*, 4(84).

62 McCalman J, Bainbridge R, Percival N, et al. 2016, The effectiveness of implementation in Indigenous Australian healthcare: An overview of literature reviews, *Int J Equity Health*, 15(47).

63 Ralph AP, Read C, Johnston V, et al. 2016, Improving delivery of secondary prophylaxis for rheumatic heart disease in remote Indigenous communities: Study protocol for a stepped-wedge randomised trial, *Trials*, 17(51).

64 Searles A, Doran C, Attia J, et al. 2016, An approach to measuring and encouraging research translation and research impact, *Health Res Policy Syst*, 14(1).

65 Hayward MN, Mequanint S, Paquette-Warren J, et al. 2017, The FORGE AHEAD clinical readiness consultation tool: A validated tool to assess clinical readiness for chronic disease care mobilization in Canada's First Nations, *BMC Health Serv Res*, 17(1).

66 Laycock A, Bailie J, Matthews V, et al. 2017, A developmental evaluation to enhance stakeholder engagement in a wide-scale interactive project disseminating quality improvement data: Study protocol for a mixed-methods study, *BMJ Open*, 7(7).

67 Meiklejohn JA, Garvey G, Bailie R, et al. 2017, Follow-up cancer care: Perspectives of Aboriginal and Torres Strait Islander cancer survivors, *Support Care Cancer*, 25(5):1597.

68 Ramanathan S, Reeves P, Deeming S, et al. 2017, Encouraging translation and assessing impact of the Centre for Research Excellence in Integrated Quality Improvement: Rationale and protocol for a research impact assessment, *BMJ Open*, 7(12).

69 Bailie J, Boyle J & Bailie R. 2018, Population attributable fractions of perinatal outcomes for nulliparous women associated with overweight and obesity, 1990–2014, *Med J Aust*, 208(11).

70 Edmond KM, McAuley K, McAullay D, et al. 2018, Quality of social and emotional wellbeing services for families of young Indigenous children attending primary care centers; a cross sectional analysis, *BMC Health Serv Res*, 18(1).

71 Heyeres M, Kinchin I, Whatley E, et al. 2018, Evaluation of a residential mental health recovery service in North Queensland, *Front Public Health*, 6(123).

72 Meiklejohn JA, Arley B, Bailie R, et al. 2018, Community-identified recommendations to enhance cancer survivorship for Aboriginal and Torres Strait Islander people, *Aust J Prim Health*, 24(3):233–40.

73 Onnis L-a, Klieve H & Tsey K 2018, The evidence needed to demonstrate impact: A synthesis of the evidence from a phased social and emotional wellbeing intervention, *Eval and Program Plann*, 70:35–43.

74 Onnis L-a, Tsey K, Hakendorf M, et al. 2018, Can integrating workplace health and wellbeing initiatives into existing leadership programs provide a sustainable solution for improving the health, wellbeing and performance of managers?, paper presented at Academy of Management Conference, Australian and New Zealand Academy of Management, Auckland.

75 Ralph AP, de Dassel JL, Kirby A, et al. 2018, Improving delivery of secondary prophylaxis for rheumatic heart disease in a high-burden setting: Outcome of a stepped-wedge, community, randomized trial, *J Am Heart Assoc*, 7(14).

76 Strobel NA, McAuley K, Matthews V, et al. 2018, Understanding the structure and processes of primary health care for young indigenous children, *J Prim Health Care*, 10(3):267–78.

77 Adily A, Girgis S, Matthews V, et al. 2019 [in press], Syphilis testing performance in Aboriginal primary health care: Exploring impact of continuous quality improvement over time, *Aust J Prim Health*.

78 Carrington A, Dewar S, Kinchin I, et al. 2019, A police-led community response to child abuse and youth sexual violence and abuse in Indigenous communities in Far North Queensland: 'Speak Up. Be strong. Be Heard', *Child Abuse Negl*, 98(104228).

79 Carroll SJ, Dale MJ, Bailie R, et al. 2019, Climatic and community sociodemographic factors associated with remote Indigenous Australian smoking rates: An ecological study of health audit data, *BMJ Open*, 9(7).

80 Conte KP, Gwynn J, Turner N, et al. 2019, Making space for Aboriginal and Torres Strait Islander community health workers in health promotion, *Health Promot Int*, pii: daz035.

81 Cunningham FC, Ranmuthugala G, Westbrook JI, et al. 2019, Tackling the wicked problem of health networks: The design of an evaluation framework, *BMJ Open*, 9(5).

82 Fazelipour M & Cunningham F 2019, Barriers and facilitators to the implementation of brief interventions targeting smoking, nutrition, and physical activity for indigenous populations: A narrative review, *Int J Equity Health*, 18(1):169.

83 Heyeres M, Tsey K, Yang Y, et al. 2019, The characteristics and reporting quality of research impact case studies: A systematic review, *Eval and Program Plann*, 73:10–23.

84 Kinchin I, Russell AMT, Tsey K, et al. 2019, Psychiatric inpatient cost of care before and after admission at a residential subacute step-up/step-down mental health facility, *J Med Econ*, 22(5):491–8.

85 Laycock A, Bailie J, Matthews V, et al. 2019, Using developmental evaluation to support knowledge translation: reflections from a large-scale quality improvement project in Indigenous primary healthcare, *Health Res Policy Syst*, 17(70).

86 Lopez-Carmen V, McCalman J, Benveniste T, et al. 2019, Working together to improve the mental health of indigenous children: A systematic review, *Child Youth Serv Rev*, 104(104408).

87 Preston R, Rannard S, Felton-Busch C, et al. 2019, How and why do participatory women's groups improve the quality of maternal and child health care? A systematic review protocol, *BMJ Open*, 9(9).

88 Quinn E, Girgis S, Van Buskirk J, et al. 2019, Clinic factors association with better delivery of secondary prophylaxis in ARF management, *Aust J Gen Pract*, 48(12):859–865

89 Valery PC, Bernardes CM, de Witt A, et al. 2020, Patterns of primary health care service use of Indigenous Australians diagnosed with cancer, *Support Care Cancer*, 28(1):317–27.

90 Zuchowski I, Miles D, Gair S, et al. 2019, Social work research with industry: A systematic literature review of engagement and impact, *Br J Soc Work*, bcz015.

e-Books

91 Bailie R, Larkins S & Broughton E (eds) 2017, *Continuous Quality Improvement – Advancing Understanding of Design, Application, Impact and Evaluation of CQI Approaches*, Research Topic eBook, Frontiers Media, Lausanne, Switzerland.

92 Tsey K 2019, *Working on Wicked Problems: A Strengths-based Approach to Research Engagement and Impact*, Springer Nature, Switzerland.

The Visual Bibliography and List of References are part of the final report of the CRE-IQI.

Laycock, A., Conte, K., Harkin, K., Bailie, J., Matthews, V., Cunningham, F. Ramanathan, S. & Bailie, R. 2020, *Improving the Quality of Primary Health Care for Aboriginal and Torres Strait Islander Australians. Centre for Research Excellence in Integrated Quality Improvement 2015–2019: Messages for Action, Impact and Research*, University Centre for Rural Health, The University of Sydney, Lismore, NSW.

CRE-IQI
Centre for Research Excellence in Integrated Quality Improvement
an Innovation Platform in Indigenous Primary Health Care
